# Spectroscopic Behavior and Photophysical Parameters of 2-(Acetoxymethyl)-6-(1,2,4-triazinylaminodihydroquinazolinyl)tetrahydropyran Derivative in Different Solid Hosts

**DOI:** 10.1007/s10895-022-02960-z

**Published:** 2022-05-06

**Authors:** Mahmoud E. M. Sakr, Maram T. H. Abou Kana, Ahmed H. M. Elwahy, Mohamed S. Abdelwahed, Samy A. El-Daly, El-Zeiny M. Ebeid

**Affiliations:** 1 Laser Sciences and Interactions Department, National Institute of Laser-Enhanced Sciences (NILES), Cairo University, Giza, Egypt; 2 Chemistry Department, Faculty of Science, Cairo University, Giza, Egypt; 3 Chemistry Department, Faculty of Science, Tanta University, Tanta, Egypt; 4 Misr University for Science and Technology (MUST), 6th of October City, Egypt

**Keywords:** Optical property, Solid state laser dye, Photophysical parameters, Photostability, Silicate based sol–gel, Polymeric matrices

## Abstract

Optical and photophysical properties of 6-substituted-1,2,4-Triazine fluorescent derivative dye doped in silicate based sol–gel, homopolymer of methyl methacrylate (PMMA), and copolymer (MMA/diethylene glycol dimethacrylate) (DEGDMA) were investigated. The pores of different hosts and caging of the dye were found to effect on the parameters such as molar absorptivity, cross sections of singlet–singlet electronic absorption and emission spectra, excited state lifetime, quantum yield of fluorescence. The dipole moment of electronic transition, the length of attenuation and oscillator strength of electronic transition from So → S1 have been calculated. The dye was pumped with different powers using 3^rd^ harmonic Nd: YAG laser of 355 nm and pulse duration 8 ns, with repetition rate 10 Hz. Good photo stability for dye was attained. After 55,000 pumping pulses of (10 mJ/pulse), the photo-stabilities were decreased to 53%, 48%, and 45% of the initial ASE of dye in sol gel, PMMA, and Co-polymer respectively. The dye in sol–gel matrix showed improvement of photo stability compared with those in organic polymeric matrices.

## Introduction

1,2,4-triazine is an important core system and many of their derivatives have gained considerable attention because they are found in numerous natural and synthetic biologically as well as pharmacologically active compounds [1]. 1,2,4-Triazine derivatives have been reported to possess a broad spectrum of biological activities including anti-inflammatory [2] antimicrobial [3, 4], anti-HIV [5], anticancer [6–8], anti haemostatic activity [9], antiviral [10], anti-malarial [11], anticonvulsant [8], neuro protective [12], antifungal [13], anti-proliferation [14]. Some 1,2,4-triazine derivatives have also used as kinase inhibitors [15], and α-glucosidase inhibitors [16]. Also fused heterocyclic systems that contain nitrogen were reported to exhibit fluorescent activity [17]. They are also widely applied as LEDs, lasers of semiconductors, probes, and fluorescent sensors. In the development of organic LED (OLED) technologies trends are focused primarily on optimizing existing devices and developing new emission materials [18, 19]. In recent years, the synthesis of new high-performance dyes and the implementation of new ways of incorporating the organic molecules into the solid matrix have resulted in significant advances towards the development of practical tunable solid-state dye lasers, due to their high efficiency and they do not contain volatile and toxic solvents, they are non-flammable, nontoxic, compact in size and mechanically and thermally more stable [20–23]. The sol–gel method is a method in which organic dye molecules are incorporated into an inorganic silica host [24]. This shows some advantages, e.g. compactness, better manageability and highly porous, transparent in Uv–visible-near IR regions. Its reaction can be controlled easily by chemical methods. It allows introducing permanent organic groups to form inorganic–organic hybrid materials [25] and the process takes place at low temperature [26]. In this respect we recently reported the synthesis of 6-Substituted-1, 2, 4-Triazine mono glucosyl fluorescent derivative dye and investigated their optical, photo physical and solvatochromic properties [27]. In continuation of this work, the present study discuss the spectral behavior and photophysical parameters of 6-substituted-1,2,4-triazine mono glucosyl fluorescent derivative dye doped in various solid hosts matrices including sol–gel, PMMA and Co-PMMA. Although a lot of dyes are commercially available for laser systems, but the previous ones have a pointing of advantages such as larger Stokes shift magnitude (**Δλ > **100 nm) which can minimize cross-talk between the excitation source and the fluorescent emission [28].

## Experimental

### Materials

Active triazine derivative as chromophore was recently prepared and reported in described form by our lab’team [27]
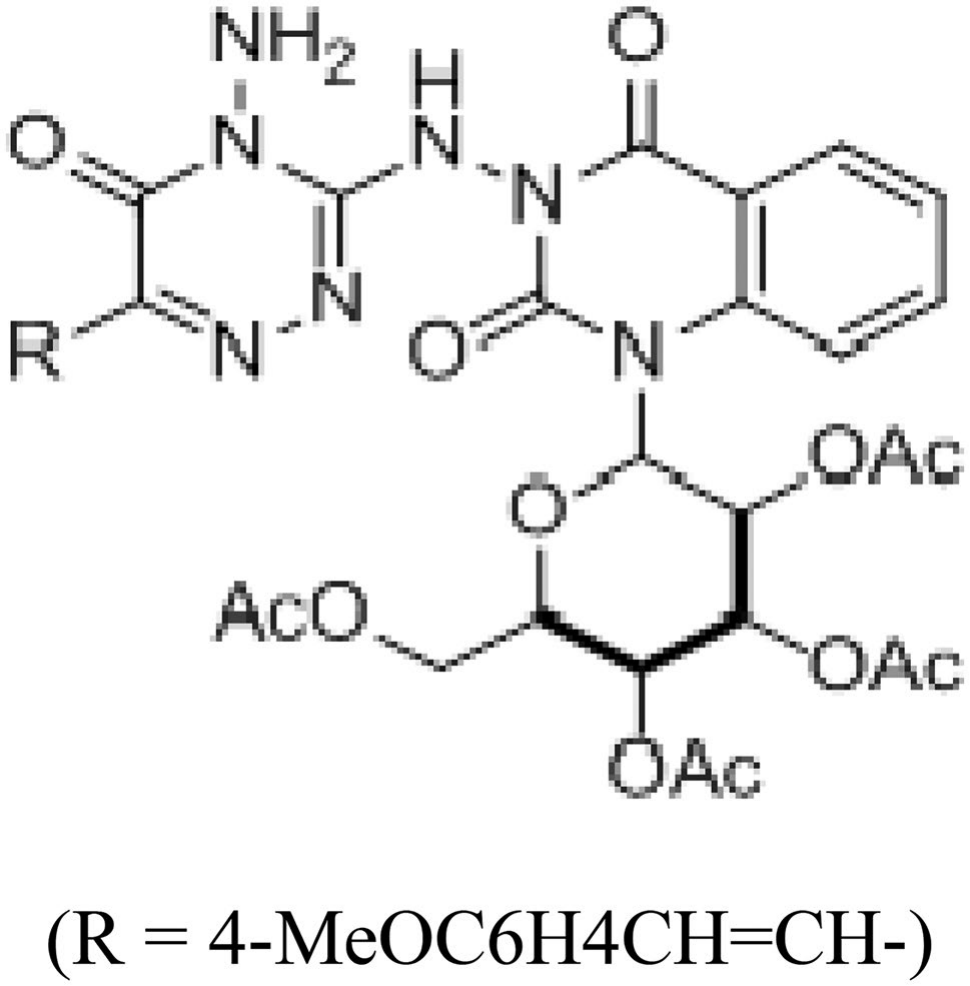


Solid hosts as; silicate based sol–gel matrix and polymer (using methyl methacrylate MMA and diethylene glycol dimethacrylate DEGDMA as monomers) were prepared as described in references [28] and [29, 30], respectively.

### Spectral Measurements

Dye samples of concentrations ranging from 2 × 10^–5^ M to 3 × 10^–4^ M were doped in transparent sol–gel, PMMA and (MMA / DEGDMA) Co-polymer. The electronic absorption properties of the dye samples in different solid hosts were studies using a Camspec M501 UV–Vis spectrophotometer. The emission spectra were monitored, depend upon exciting wavelength which represent maximum absorption, using JASCO-spectrofluorometer (model: PF-6300). Laser induced fluorescence of dye samples as function of different input pumping energies were carried out by 3^rd^ harmonic Nd: YAG laser using homemade setup as previously reported [31]. The photostability of the dye was also determined [32]. The input energy was kept constant at (10 mJ) by pumping with 355 nm of 3rd harmonic Nd: YAG laser.

### PhotoPhysical Parameters Calculations

Depending upon these absorption and emission spectra, important and significant photophysical parameters could be determined according to their standard equations as previously reported such as: the oscillator strength [33], the attenuation length $\Lambda (\lambda )$ [34], the dipole moment transition μ_12_ [34], the rate of radiative decay constant (kr) [35], the absorption cross-section σ_a_ [36], the quantum yield (ϕ_f_) of a compound relative to a standard probe [37, 38], fluorescence lifetimes (τ_f_) [39], the rate constant of intersystem crossing (kisc) which related to the quantum yield ϕ_f_ for (ϕf ≈1) by the relationship (1) [35]:

1 $${\mathrm{k}}_{\mathrm{isc}}=\left(1-{\phi }_{\mathrm{f}}\right)/{\tau }_{\mathrm{f}}$$

Also, the emission cross-section σ_e_ was calculated according to ref. [40].

## Results and Discussion

### Photphysical Properties in Different Hosts

**Fig. 1:**
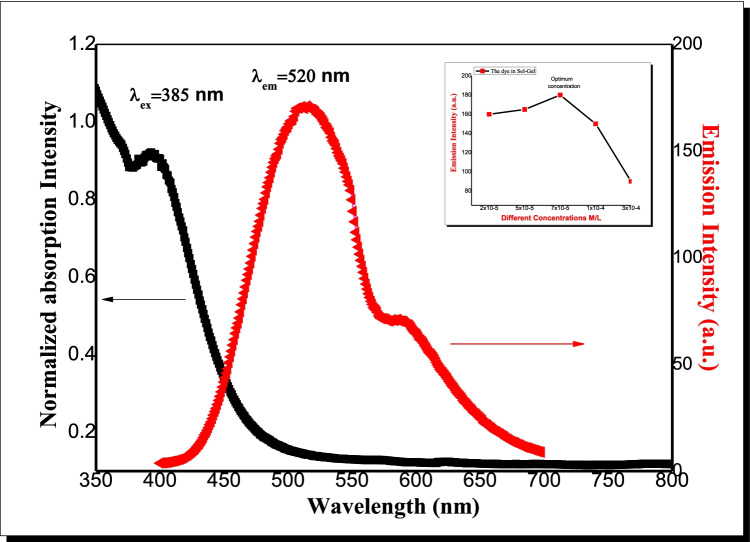
Normalized absorption spectra and the emission spectra of dye of 7 × 10^–5^ M with excited wavelength 385 nm in sol–gel (the inset figure shows the dye emission intensity as function of different concentrations)

**Fig. 2:**
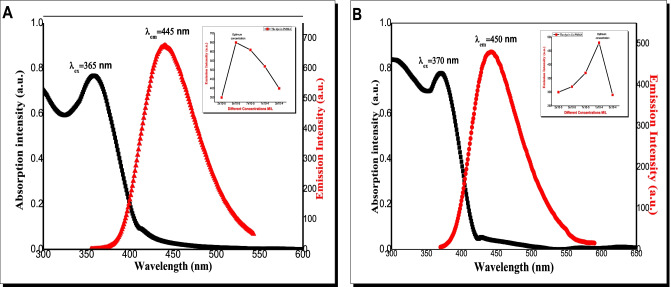
(**A**, **B**) The absorption and emission spectra of 5 × 10^–5^ M dye in **A**) PMMA and **B**) (MMA/DEGDMA) Co-polymer with excited wavelength 365 nm and 370 nm, respectively. (The inset figures focus on the dye emission intensity as function of different concentrations)

The UV–visible absorption and fluorescence spectra of the dye in sol–gel, homo-polymer PMMA and (MMA / DEGDMA) copolymer matrices as solid hosts are shown in Figs. 1 and 2. There is a minimum overlap between the dye absorption and emission spectra in the three solid matrices. This is important as far as reabsorption of emitted photons is concerned. Figure 1 shows that the absorption maximum peak of dye was 385 nm and the emission maximum peak was at 520 nm in sol–gel, respectively. Inset Fig. 1 shows that the emission of the dye of different concentrations in sol–gel at excitation wavelengths 385 nm. The optimum dye concentration was 7 × 10^–5^ M in sol–gel. The dye fluorescence peak intensity increased till 7 × 10^–5^ M then decreased with increasing concentrations which might be attributed to due molecular aggregations of dye molecules which absorbed the emitted photon.

Figure 2A, B shows that the absorption maximum peaks were at 365 and 370 nm in PMMA and (MMA/DEGDMA), respectively. It also shows that the emission maximum peaks were at 445 nm and 450 nm for the dye in PMMA and (MMA/DEGDMA), respectively. Inset Fig. 2A, B shows that the emission of the dye at different concentrations in in PMMA and (MMA/DEGDMA) at excitation wavelength 365 nm, and 370 nm respectively. The dye emission spectra with different concentrations doped in PMMA and (MMA/DEGDMA) Co-polymer was shown in inset Fig. 2A, B. The dye fluorescence peak intensity increased till 5 × 10^–5^ M in case of PMMA and 1 × 10^–4^ in case of (MMA/DEGDMA) Co-polymer then it decreased with increasing concentrations which might be attributed to molecular aggregations of dye molecules. Also, the optimum dye concentration was 5 × 10^–5^ M and 1 × 10^–4^ M in PMMA and (MMA/DEGDMA) Co-polymer, respectively.

From the Figs. 1 and 2 show that the absorption maximum peaks were 385 nm, 365 nm and 370 nm in sol gel, PMMA and (MMA/DEGDMA), respectively, and the emission maximum peaks were 520 nm, 445 nm and 450 nm in sol gel, PMMA and (MMA/DEGDMA), respectively. We found that the nature of polymer either homo-polymer or copolymer has no effect on the absorption intensity of the dye, but it has clear effect of its absorption wavelength. This effect may be attributed to the nature of interaction between dye and DEGDMA which contains different active groups. Comparing the dye fluorescence peak wavelengths and intensities in different solid matrices showed that, the fluorescence emission wavelengths are higher red shifted in sol–gel compared to polymer matrices. This behavior indicates a more relaxed excited state due to dye host interaction within excited state lifetime. Some important photo-physical parameters of the dye were calculated and summarized in Table 1, which demonstrate their potential for use in advanced optical applications. However, the absorption cross-section (σa) is the ability of the molecule to absorb a photon of a certain polarization and wavelength. Emission cross-section (σe) measures the probability of the excited ion in a given cross sectional area to emit a photon. The attenuation length (L) (called absorption length) is the distance into a material when the probability has dropped to1/*e* that a particle has not been absorbed.

**Table 1:** Photophysical parameters of the dye in different hosts; (ε) molecular extinction coefficient; σa and σe: cross section of absorption and emission; (Λ) the attenuation length, (τf) fluorescence life time, (τo) calculated fluorescence life time, μ12(D) the dipole moment transition, (Ef) energy yield of fluorescence, (Kr) the radiative decay rate, (Kisc) the rate of intersystem crossing, (f) oscillator strength, φf fluorescence quantum yield, in different hosts

**Sample /matrix**	**ε** **L.M**^**−1**^**.Cm**^**−1**^** (10**^**4**^**)**	**σ**_**a**_ **(10**^**–16**^**) Cm**^**2**^	**σ**_**e**_ **(10**^**–17**^**) Cm**^**2**^	**Λ (cm)**	**τ**_**f**_ **(ns)**	**τ**_**0**_ **(1/k**_**r**_**)** **(ns)**	**μ**_**12**_ **(D)**	**E**_**f**_	**K**_**r**_ **(10**^**9)**^ **s**^**−1**^	**K**_**isc**_ **(10**^**9**^**)** **s**^**−1**^	**F**	**φ**_**f**_
**Solgel**	**1.5**	**0.6**	**2,2**	**0.2**	**0.6**	**0.9**	**3.53**	**0.31**	**1.1**	**0.9**	**1.74**	**0.7**
**PMMA**	**3.5**	**1.4**	**2.6**	**0.1**	**0.2**	**0.4**	**3.35**	**0.28**	**2.8**	**1.8**	**1.33**	**0.6**
**(MMA/DEGDMA)**	**3.5**	**1.3**	**2.4**	**0.1**	**0.2**	**0.4**	**2.96**	**0.23**	**2.6**	**1.6**	**1.02**	**0.5**

It is noticed from photophysical parameters of dye in different solid hosts that excited state lifetime (τ_f_) values in PMMA and in copolymer matrices are lower than those in sol–gel matrix. This indicates a dynamic quenching process in which the polymer matrices interact with the excited state dye molecules. This leads to shortening of τ_f_ values in polymer matrices compared with sol–gel. The oscillator strength value in sol–gel matrix is higher than those in polymer matrices. Hence, the effective number of electrons transferred from the ground to excited states in sol–gel is higher than that in polymer matrices. Fluorescence quantum yield (ϕ_f_) values are lower in polymer and in copolymer matrices compared with those in sol–gel, indicating more interaction between dye molecules and polymer matrices. The carbonyl group in chromophore polymer matrices possesses (n, π*) electronic states that are characterized by low singlet—triplet splitting energies (ΔES,T) leading to triplet state population from singlet excited state, with subsequent fluorescence quenching [34]. It is known that the (T1 → Tn) transition is a spin- allowed one that can quench fluorescence by photon re-absorption. The lower energy level of (n, π*) states also allows for exciton trapping [34] adding to factors causing fluorescence quenching. Further confirmation of the role of polymer matrices in fluorescence quenching comes from the higher intersystem crossing rate constants (k_isc_) values in polymer matrices compared with sol–gel glass.

### Laser-induced Fuorescence of 6-substituted-1,2,4-triazines Mono Glucosyl Derivative

**Fig. 3:**
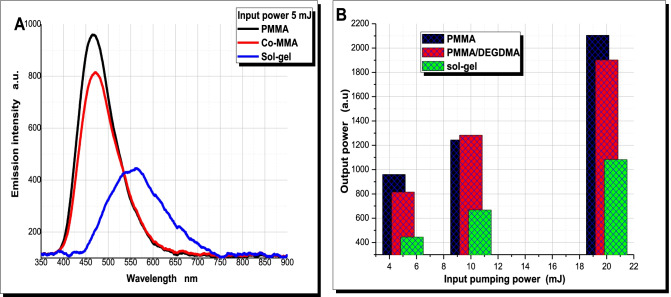
(**A**, **B**): **A**) Emission intensities at input pumping power 5 mJ, and **B**) The output powers at different input pumping powers 5 mJ, 10 mJ, 20 mJ of the dye in PMMA, Co-PMMA(MMA/DEGDMA), and sol–gel with excited wavelength 355 nm by 3rd^rd^ harmonic Nd:YAG pulsed laser

The spontaneous fluorescence intensities and wavelengths of the 6-substituted-1,2,4-triazines mono glucosyl derivative dye in sol gel, PMMA and (MMA/DEGDMA) copolymer were varied after pumping with 3^rd^ harmonic Nd:YAG (λ = 355 nm). Emission intensity of ASE of the dye with concentrations range from 2 × 10^–5^ M to 3 × 10^–4^ M in PMMA, Co-PMMA (MMA/DEGDMA), and sol–gel with excited wavelength 355 nm by 3rd^rd^ harmonic Nd: YAG pulsed laser at pumping power 5 mJ. We found that the concentration 3 × 10^–4^ after pumping the different concentration by 5 mJ is the highest emission intensity of the dye in PMMA, Co-PMMA (MMA/DEGDMA) and sol–gel then pumping these concentration 3 × 10^–4^ with excitation wavelength 355 nm by 3rd harmonic Nd:YAG pulsed laser with different input pumping powers 5 mJ, 10 mJ, 20 mJ as showed in Fig. 3A−C.

By pumping using 355 nm 3rd^rd^ harmonic Nd: YAG pulsed laser with λ_ex_ = 355 nm excitation wavelength at different powers intensity 5 mJ, 10 mJ, and 20 mJ. The emission intensity of ASE peak wavelength of the dye in sol gel matrix (λ_f_ = 550 nm) showed a large red shift from that of the dye in PMMA (λ_f_ = 475 nm) and (MMA/DEGDMA) matrix (λ_f_ = 480 nm) as in Fig. 3. Figure 3 showed the emission intensity of the dye in solid hosts at different input power at 5, 10 and 20 mJ. The increasing of the peak intensity of ASE of the dye may be attributed to the increasing of the number of excited molecules (increase the population of S1 state) which yields more emitted photons. These changes in wavelengths may be due to the interaction of the structure of the two different dyes molecules with different solid matrices as shown in Fig. 3.

**Fig. 4:**
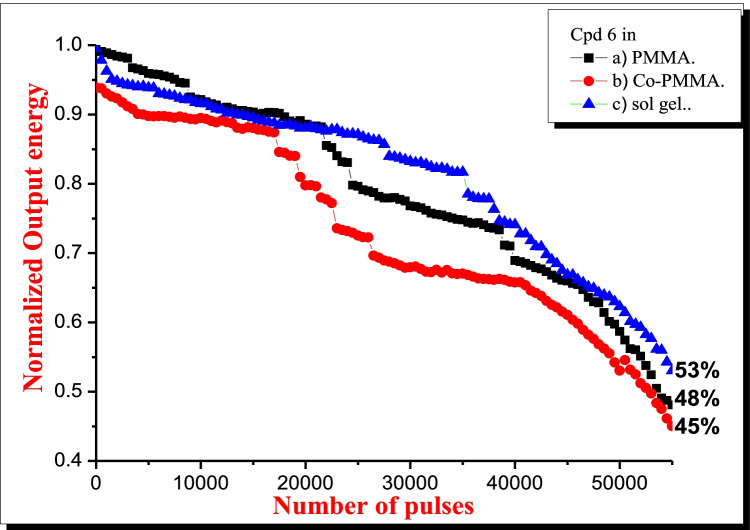
Normalized output energy of the dye as a function of the number of pump pulses using pumping power of 355 nm 3rd harmonic Nd: YAG laser at 10 mJ

The photostability, as an important photochemical parameter, was studied by the evaluation of the output fluorescence as a function of number of pulses in the same position of the samples as outlined in Fig. 4. This study was carried out for the samples of the dyes which the repetition rate of 355 nm Nd: YAG laser with 8 ns pulse duration was kept at (10 Hz) and the energy was kept constant at (10 mJ/pulse). The output energy gradually decreased due to the photodegradation progressive and thermo-degradation of the dye’s molecules. This decreasing occurred at a faster rate for the dyes in polymer than in sol gel, and the peak ASE dropped to 53%, 48% and 45% of the initial ASE of the dye in sol–gel, PMMA and (MMA/DEGDMA) Co-PMMA, respectively, by pumping with 355 nm 3^rd^ harmonic Nd: YAG laser at 10 mJ with repetition rate of 10 Hz after 55,000 pulses. Since the mechanism of photodegradation occurs by the interaction molecules of the dye in the excited state with other species such as impurities, other dye molecules and singlet oxygen. Through the process of doping dyes into a solid medium, the photochemical pathways including bimolecular reactions can be suppressed by caging or trapping the dye within a solid host [41].

The improved photostability of the dyes within solid hosts has been attributed for caging and molecules immobilizing of the dye, minimizing thereby excited-state interaction with other species including molecular oxygen. So, the dye photodegradation in a solid host depends on the dye’s molecule nature, the host composition and structure, the host impurities as well as presence of molecular oxygen. Another possible factor that may explains the reduced rate of degradation in the rigid matrices may be that the dyes molecules are more tightly confined within the pores of the matrix, limiting rotational and translational freedom. A mobile molecule, as in solution, will be more likely to encounter an oxygen molecule and undergo degradation. Less freedom, as defined by the restrictive pores of the matrix, may make the dye molecules less likely to interact with molecular oxygen leading to photodegradation or molecular oxygen fluorescence quenching [41]. The micro-viscosity environment around dye molecules in the solid matrix affects their photodegradation. The net photo deterioration would be slow if the irradiated molecules were swiftly replaced by fresh molecules. As a result, photo degradation in sol–gel samples is negligible, with the longest half-life values. On the other hand, photo degradation occurs at a faster pace in the copolymer samples. This is because dye molecules in polymer samples are surrounded only by polymer matrix with very little solvent around them, whereas dye molecules in sol–gel matrix are rapidly replenished because sol–gel samples contain ethylene glycol, which may aid in the mobility of the embedded dye molecules, resulting in minimal photodegradation and the longest half-life values.

## Conclusion

The optical absorption and emission properties of 2-(acetoxymethyl)-6-(1,2,4-triazinylaminodihydroquinazolinyl)tetrahydropyran dye have been studied in different solid hosts such as sol–gel, PMMA and DEGDMA copolymer. Their respective spectroscopic and photophysical parameters meet the best requirements for a good laser dye such as high molar absorption coefficients at the wavelength of the pump laser, broad spectral region of fluorescence and high fluorescence quantum yield, short fluorescence decay time, large Stokes' shift, little overlap of the fluorescence and triplet absorption spectral regions, photochemical stability. Pumping the samples using 3^rd^ harmonic Nd: YAG laser (λ_ex_ = 355 nm) showed different emission wavelength of ASE peak. It was nearly in sol gel matrix (λ_f_ = 550 nm), in PMMA (λ_f_ = 475 nm) and (MMA/DEGDMA) copolymer (λ_f_ = 480 nm). The nature of solid host has significant effect on spectroscopic properties of dye. The new dye exhibited good photostability. It decreased to 53%, 48% and 45% of the initial ASE of the dye in sol–gel, PMMA and DEGDMA copolymer, respectively, after pumping with 355 nm 3^rd^ harmonic Nd: YAG laser of 8 ns pulse duration, with a repetition rate (10 Hz). The energy was kept constant at (10 mJ/pulse) after 55,000 pulses.

## Data Availability

Data generated or analyzed during this study are included in this published article.
